# Conceptual Design of a Novel Nozzle Combined with a Clinical Proton Linac for Magnetically Focussed Minibeams

**DOI:** 10.3390/cancers13184657

**Published:** 2021-09-16

**Authors:** Tim Schneider, Annalisa Patriarca, Alberto Degiovanni, Manuel Gallas, Yolanda Prezado

**Affiliations:** 1 Institut Curie, Université Paris Saclay, CNRS UMR3347, Inserm U1021, Signalisation Radiobiologie et Cancer, 91400 Orsay, France; yolanda.prezado@curie.fr; 2 Centre de Protonthérapie d’Orsay, Radiation Oncology Department, Institut Curie, PSL Research University, 91898 Orsay, France; annalisa.patriarca@curie.fr; 3 ADAM SA, 1217 Meyrin, Switzerland; alberto.degiovanni@avo-adam.com (A.D.); manuel.gallas@avo-adam.com (M.G.)

**Keywords:** minibeam radiation therapy, protons, pMBRT, linac, magnetic focussing, Monte Carlo, clinical minibeams

## Abstract

**Simple Summary:**

Proton minibeam radiation therapy (pMBRT) is a novel therapeutic strategy that combines the tissue sparing potential of submillimetric, spatially fractionated beams (minibeams) with the improved ballistics of protons to enhance the tolerance of normal tissue and allow a dose escalation in the tumour. This approach could allow a more effective treatment of radioresistant tumours and has already shown excellent results for rat gliomas. To exploit the full potential of pMBRT, it should be delivered using magnetically focussed and scanned minibeams. However, such an implementation has not yet been demonstrated at clinically relevant beam energies. In this work, we therefore present a new design combining our recently developed minibeam nozzle with the first clinical proton linear accelerator. We show the suitability of this combination for the generation of magnetically focussed and scanned minibeams with clinically relevant parameters as well as for the delivery of conventional pencil beam scanning techniques.

**Abstract:**

(1) Background: Proton minibeam radiation therapy (pMBRT) is a novel therapeutic approach with the potential to significantly increase normal tissue sparing while providing tumour control equivalent or superior to standard proton therapy. For reasons of efficiency, flexibility and minibeam quality, the optimal implementation of pMBRT should use magnetically focussed minibeams which, however, could not yet be generated in a clinical environment. In this study, we evaluated our recently proposed minibeam nozzle together with a new clinical proton linac as a potential implementation. (2) Methods: Monte Carlo simulations were performed to determine under which conditions minibeams can be generated and to evaluate the robustness against focussing magnet errors. Moreover, an example of conventional pencil beam scanning irradiation was simulated. (3) Results: Excellent minibeam sizes between 0.6 and 0.9 mm full width at half maximum could be obtained and a good tolerance to errors was observed. Furthermore, the delivery of a 10 cm × 10 cm field with pencil beams was demonstrated. (4) Conclusion: The combination of the new proton linac and minibeam nozzle could represent an optimal implementation of pMBRT by allowing the generation of magnetically focussed minibeams with clinically relevant parameters. It could furthermore be used for conventional pencil beam scanning.

## 1. Introduction

Proton minibeam radiation therapy (pMBRT) is a novel therapeutic strategy that combines the normal tissue sparing of submillimetric, spatially fractionated beams with the improved dose deposition of protons [1]. In contrast to conventional active scanning approaches where comparatively large beam spots (with diameters of five millimetres to several centimetres) are positioned to overlap at the skin level, pMBRT uses submillimetric beams (so-called *minibeams*) which are spaced apart at the skin level in order to create a distinct spatial modulation of the dose consisting of alternating regions of high dose (peaks) and low dose (valleys). This spatial fractionation can increase the tolerance of normal tissue and may allow a safe dose escalation in the tumour [2,3].

The optimal generation of proton minibeams remains an important challenge on the way towards clinical applications. Recent implementations of pMBRT at clinically relevant energies relied on mechanical collimators attached to the nozzle exit [4,5,6] or positioned a few centimetres upstream of the irradiation target [7]. While this approach is straightforward and in principle readily implementable at any existing facility, it comes at the cost of a low efficiency (due to a considerable reduction of the dose rate) and a poor flexibility (custom collimators may have to be fabricated for each patient or patient group). Furthermore, the collimator represents a source of unwanted secondary particles such as neutrons.

An approach to overcome these limitations would be the use of magnetically focussed and scanned minibeams for pMBRT. Indeed, recent publications [8,9] suggest that the optimal implementation of pMBRT should use magnetic focussing instead of mechanical collimation for minibeam generation, as it can significantly increase both the irradiation efficiency and flexibility and also improve the degree of spatial fractionation in healthy tissue.

While this method is conceptually very similar to the established pencil beam scanning (PBS) techniques, a crucial difference lies in the beam sizes used in the two cases: Beams used for PBS typically have a full width at half maximum (FWHM) between 5 and 20 mm at the isocentre [10,11,12] whereas minibeams should have a FWHM≤1 mm (at the skin level) and a small divergence (≲10 mrad) to ensure the optimal exploitation of tissue sparing effects. Conventional PBS nozzles will likely not be able to provide magnetically focussed minibeams due to a too long focal length and the presence of too much air in the beam path and thus a new, improved nozzle design was developed [13].

Besides the geometry of the nozzle, another crucial factor for the generation of minibeams is the beam entering the nozzle. In particular, the beam should have a small emittance as well as a small divergence or an extreme correlation between the spatial and angular spread of the beam particles [13]. While it is very likely that contemporary cyclotron systems used for proton therapy will not be suitable for the generation of such beams [14], new approaches like medical linear accelerators appear very promising. In this work, we therefore considered the practical feasibility of using the new minibeam nozzle design in combination with the linear accelerator LIGHT (Linac For Image Guided Hadron Therapy) [15,16].

The LIGHT accelerator is the first high frequency linear accelerator for proton therapy working at 3 GHz designed as an industrial product by Advanced Oncotherapy (AVO) and its subsidiary Application of Detectors and Accelerators to Medicine (ADAM). The AVO-ADAM linac design consists of three different linac sections: Firstly, a radio frequency quadrupole (RFQ) used as the injector accelerating the beam up to 5 MeV; secondly, a side coupled drift tube linac (SCDTL) section, that accelerates the beam from 5 to 37.5 MeV; thirdly, a cell coupled linac (CCL) section bringing the beam to its final energy of 230 MeV.

The high frequency linac modules of SCDTL and CCL are powered independently by 3 GHz klystrons. This modularity allows to achieve variable energy beams at the end of the CCL cavities. The beam energy can be actively modulated between 70 and 230 MeV without the need for an absorber or degrader. Furthermore, the linac approach, without the need of complex injection and extraction system, allows to accelerate the beam with a much smaller emittance compared to other proton therapy systems which makes it possible to use small aperture magnets for the transfer lines and gantry.

In this study, the performance of the new minibeam nozzle design in combination with the LIGHT accelerator was evaluated. The main focus lay on the generation of magnetically focussed and scanned proton minibeams (here we consider a beam to be a *minibeam* when the horizontal or vertical FWHM is ≤1 mm) and their robustness to various errors affecting the focussing magnets. Beyond that, the compatibility of the new nozzle for the delivery of conventional PBS fields was also assessed. This manuscript summarises the first conceptual design demonstrating the feasibility of this implementation.

## 2. Materials and Methods

Monte Carlo simulations were performed with the Geant-4 based toolkit TOPAS (http://www.topasmc.org) [17,18] (version 3.6.p1) which is frequently used in the context of proton therapy [19,20,21,22,23,24]. The physics list was built using the *Geant4_Modular* option with the recommended modules for proton therapy (*g4em-standard_opt4*, *g4h-phy_QGSP_BIC_HP*, *g4decay*, *g4ion-binarycascade*, *g4h-elastic_HP* and *g4stopping*) [24,25,26] and the range cut was 0.05 mm in all volumes and for all particles. Two clinically relevant proton beam energies (100 and 200 MeV) were considered.

The study was divided into two parts:

(1) **Minibeam generation:** The first part considered the generation of proton minibeams. For this, first the minimum beam size achievable at a specified target position was determined for different beam parametrisations at the nozzle entrance. The aim of this was to identify the conditions for which minibeams (i.e., beams with a FWHM≤1 mm) can be obtained. Moreover, simulations evaluating the robustness were performed which considered the effect of errors in magnet alignment and magnetic fields.
(2) **Delivery of conventional PBS irradiations:** The second part considered the delivery of conventional PBS with the new minibeam nozzle in combination with the LIGHT accelerator. For this, an example irradiation field in a water phantom was simulated.

### 2.1. Nozzle Geometry and Beam Model

**Figure 1:**
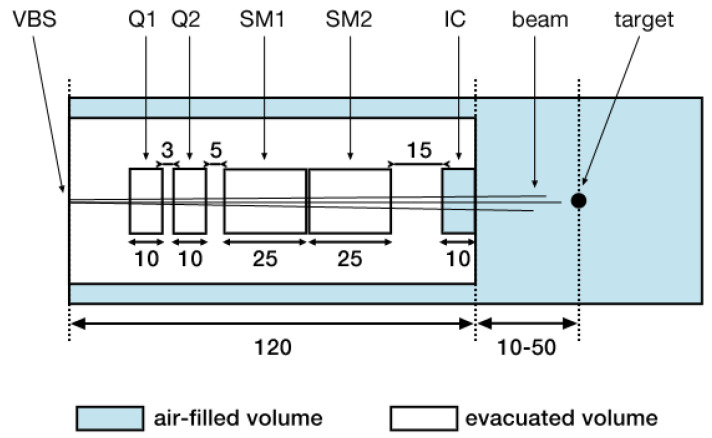
Schematic of the new nozzle design with dimensions in cm. Abbreviations: VBS - virtual beam source, Q—quadrupole, SM—scanning (dipole) magnet, IC—ionisation chamber.

The evaluated nozzle design is thoroughly discussed in a previous publication [13]. A schematic of its geometry is shown in Figure 1. The main differences compared to a conventional PBS nozzle are the shorter focal length (distance between the quadrupoles and target) and the reduction of air-filled drift spaces. Three different values of the *air gap* (the space between the exit of the ionisation chamber and the target entrance) were considered, namely 10, 30 and 50 cm.

The quadrupole magnets Q1 and Q2 were assumed to have an effective length of 10 cm and the fields were modelled using TOPAS’ *QuadrupoleMagnet* feature. The scanning magnets SM1 and SM2 had lengths of 25 cm and the dipole fields were modelled with TOPAS’ *DipoleMagnet* feature. SM1 was set to deflect the beam in the horizontal plane and SM2 scanned in the vertical plane. As in previous studies [13,14], both the quadrupole and dipole magnets used hard edge models, i.e., no fringe field effects were considered in this study. This was motivated by previous benchmarking simulations which showed no relevant differences between the beam sizes obtained with idealised quadrupole models and with detailed field maps (see chapter 4.3 in [14]).

The virtual beam source was placed at the nozzle entrance as illustrated in Figure 1. TOPAS’ *emittance* source type in *BiGaussian* distribution mode was used which generates a beam where both the spatial and angular particle distributions follow Gaussian distributions. Apart from the energy parameters, this beam model requires six transversal phase space parameters:

- The **beam size** parameters $\sigma_{x}$ and $\sigma_{y}$ which correspond to the widths of the Gaussians describing the horizontal and vertical **spatial** particle distributions, respectively.
- The **beam divergence** parameters $\sigma_{x^{\prime }}$ and $\sigma_{y^{\prime }}$ which correspond to the widths of the Gaussians describing the horizontal and vertical **angular** particle distributions, respectively.
- The **correlation** parameters $r_{xx^{\prime }}$ and $r_{yy^{\prime }}$ which correspond to the **correlation coefficients** in $xx^{\prime }$- and $yy^{\prime }$-phase space (or rather trace space), respectively.

A Gaussian distribution was also assumed for the particle energy.

The parameter values for the virtual beam source were determined from simulations of the LIGHT accelerator at beam energies of 100 and 200 MeV. In the following, this parametrisation will be referred to as *base model*. In practice, small deviations from the simulated beam parameters may be expected and in particular any changes leading to a larger beam emittance could have a negative effect on the beam focussing capabilities. Therefore, several variations of the base model were considered, corresponding to an increase of the emittance by 100% which can be expected to reasonably cover deviations from the design emittance that may arise in practice.

In terms of the previously introduced parameters, the horizontal and vertical beam emittances can be expressed as

(1) $$\varepsilon_{x}=\pi \sigma_{x}\sigma_{x^{\prime }}\sqrt{1-r_{xx^{\prime }}^{2}}\;\;\mathrm{and}\;\;\varepsilon_{y}=\pi \sigma_{y}\sigma_{y^{\prime }}\sqrt{1-r_{yy^{\prime }}^{2}}.$$

Consequently, the following four beam model variations were considered:

- **Var 1:** The **size** parameters $\sigma_{x/y}$ were increased by a factor of 2.
- **Var 2:** The **divergence** parameters $\sigma_{x^{\prime }/y^{\prime }}$ were increased by a factor of 2.
- **Var 3:** The modulus of the **correlation coefficients** was decreased such that the factors $R_{x}=\sqrt{1-r_{xx^{\prime }}^{2}}$ and $R_{y}=\sqrt{1-r_{yy^{\prime }}^{2}}$ were increased by a factor of 2. Note that the correlation coefficients reach a minimum at 0 so that the factors $R_{x/y}$ cannot be arbitrarily increased.
- **Var 4:** A **combined variation** where each of the parameters $\sigma_{x/y}$, $\sigma_{x^{\prime }/y^{\prime }}$, $R_{x/y}$ was increased by a factor of $\sqrt[{3}]{2}\approx 1.26$, resulting also in an emittance increase by a factor of 2.

Table 1 summarises the source parameters of all considered beam models.

### 2.2. Minibeam Generation

The main objective of the study was to evaluate the combination of the LIGHT accelerator and the new nozzle design for the generation of magnetically focussed proton minibeams at clinically relevant energies. For this, in a first step, the minimum beam size achievable at the target was determined for each of the beam models listed in Table 1.

As in a previous work [13], the minimisation method consisted in the simulation of different configurations of the quadrupole magnets. Concretely, 51 values for each of the field gradients in Q1 and Q2, ranging from 0 to 0.8 T/cm, were considered as well as two orientations of the focussing planes (Q1 focussing horizontally and Q2 focussing vertically and vice versa). This gives rise to 51×51×2=5202 distinct quadrupole configurations which were simulated for each of the three air gap lengths (10, 30 and 50 cm). In each case, the beam size was determined with the help of phase space files recorded at the target position. Note that this target position would correspond to the entrance of the target volume (such as a water phantom) which, however, was not included in these simulations.

The best out of the 5202 configurations was then identified by minimising the scalar quantity

(2) $$\Omega :=\sigma_{x}\sigma_{y}\left(\frac{\sigma_{x}}{\sigma_{y}}+\frac{\sigma_{y}}{\sigma_{x}}\right)=\sigma_{x}^{2}+\sigma_{y}^{2}$$

where $\sigma_{x}$ and $\sigma_{y}$ refer to the horizontal and vertical beam size at the target position, respectively. The quantity Ω takes into account the beam size as well as the shape (eccentricity) of the beam spot and therefore allows to find the symmetric minimum (see also [13]). The results of the minimisation simulations are stated with asymmetric error bars which include uncertainties related to the fit of the raw simulation data (contributing both to the upper and lower error) and uncertainties taking into account the finite step size between the considered field gradients (contributing only to the lower error).

In a second step, the tolerance of the found minibeam configurations (obtained with a 10 cm air gap) was evaluated for which the variation of the spot size and position at the target entrance position were assessed. This was done in two independent stages, each comprised of 200 simulations:

- **Translational and rotational misalignment:** For the first part, the quadrupoles Q1 and Q2 were translated laterally by an offset (dX,dY) from the beam axis. Both, dX and dY were randomly and independently sampled from a Gaussian distribution with μ=0 mm and σ=0.5 mm. Additionally, the quadrupoles were also misaligned by a horizontal tilt θ and vertical tilt φ where both angles were randomly and independently sampled from a Gaussian distribution with μ=0 deg and σ=0.5 deg.
- **Field gradient errors:** For the second part, the quadrupoles were considered to be perfectly aligned but the field gradients deviated from their nominal values $g^{0}$ by an amount dg (i.e., $g_{1}=g_{1}^{0}+dg_{1}$ and $g_{2}=g_{2}^{0}+dg_{2}$ where $g_{1}$ and $g_{2}$ are the gradients of Q1 and Q2, respctively), representing field variations caused by possible pulse-to-pulse fluctuations in the power supplies. Both, $dg_{1}$ and $dg_{2}$ were randomly and independently sampled from a Gaussian distribution with μ=0 T/cm and σ=0.01 T/cm.

In order to take into account the effects of the lateral beam scanning necessary to deliver actual treatment plans, an irradiation pattern consisting of 4 spots was simulated. In the following, the spots will be labelled as *center* (no offset from the beam axis, scanning magnets turned off), *scan y* (deviation from beam axis by 4.8/6.7 cm for 100/200 MeV, in vertical direction only), *scan x* (deviation from beam axis by 6.1/8.5 cm for 100/200 MeV, in horizontal direction only) and *scan xy* (deviation from beam axis by 4.8/6.7 cm and 6.1/8.5 cm for 100/200 MeV, along vertical and horizontal directions, respectively). The spot position as well as the horizontal and vertical size of the beam spots were assessed using phase space files recorded in air at the target entrance position.

### 2.3. Delivery of Conventional PBS Irradiations

The second part of the study investigated the delivery of conventional PBS fields with the combination of the minibeam nozzle and LIGHT accelerator. For this, the simulations performed for the beam size minimisation were reanalysed with respect to configurations yielding a target entrance beam size between 3.5 and 6 mm FWHM. Such a size should allow the delivery of laterally homogeneous dose distributions with comparatively few spots while at the same time providing very sharp penumbrae.

Good PBS configurations were found for the 50 cm air gap for which dose distributions in a water phantom (20cm×20cm×10cm at 100 MeV and 20cm×20cm×30cm at 200 MeV) were simulated. The irradiation field consisted of 21×33=693 spots for the 100 MeV beams and 21×21=441 spots for the 200 MeV beams which in both cases were laterally scanned to cover an area of 10cm×10cm at Bragg peak depth.

The dose was scored using TOPAS’ *DoseToWater* feature with a voxel size of 0.5mm×0.5mm×1mm. For each voxel, the dose uncertainty was calculated by considering the standard deviation of multiple repetitions of the simulations. Subsequently, the global relative uncertainty was computed as the root mean square of the voxel uncertainties, considering all voxels with at least half the maximum dose [27]. It was <2% in all cases.

## 3. Results

In the following, it will be convenient to speak about the *horizontal FWHM* (hFWHM) and *vertical FWHM* (vFWHM) when referring to the beam size. In the approximation of a Gaussian beam distribution, the two quantities are related to the aforementioned $\sigma_{x}$, $\sigma_{y}$ by $\mathrm{hFWHM}=2.355\;\sigma_{x}$ and $\mathrm{vFWHM}=2.355\;\sigma_{y}$.

### 3.1. Minibeam Generation

**Figure 2:**
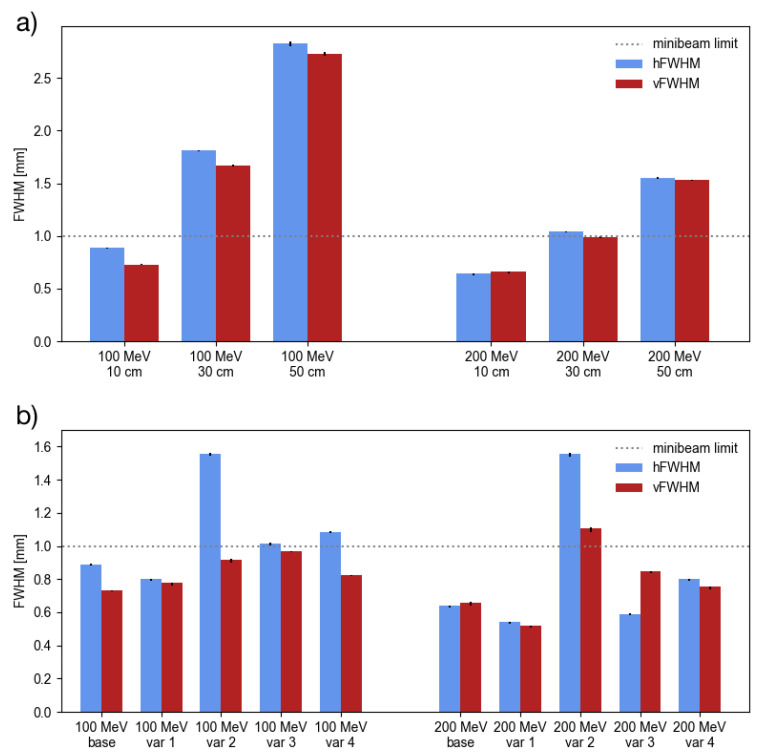
Comparison of minimum beam sizes: (**a**) For 100 and 200 MeV beams and the three different air gaps using the base beam model. (**b**) For 100 and 200 MeV beams and the four different beam model variations. The air gap was 10 cm in all cases.

Table 2 summarises the minimum beam sizes obtained with the base beam model for the three different air gaps and with the four different model variations for an air gap of 10 cm. The same beam sizes are also plotted side by side in Figure 2 to allow a more intuitive comparison.

The results show that the minimum beam size increases with the air gap which is a consequence of a longer focal length and an increased amount of multiple Coulomb scattering in air. Indeed, for a given beam parameterisation at the nozzle entrance, an approximately linear growth of the minimum beam size as a function of the focal length is expected simply from geometrical considerations (see e.g., [28]). No minibeam configuration could be obtained for the 50 cm air gap while the 30 cm air gap only allowed the generation of minibeams at 200 MeV. The best results were obtained with the 10 cm air gap allowing to generate minibeams at 200 and 100 MeV. All minibeam configurations required comparatively high field gradients ≥0.4 T/cm.

Considering the results for the varied beam models, the largest changes were observed for *var 2* where the beam divergence at the nozzle entrance was increased by a factor of 2. A substantial increase of the FWHM could be seen here, in particular concerning the hFWHM which roughly doubled and thus surpassed the minibeam limit. Less dramatic changes were observed for all other variations with beam sizes remaining close to or below the 1 mm limit. In the case of *var 1* (beam size at nozzle entrance increased by a factor of 2), it was even possible to further decrease the symmetric minima. This can be explained by the fact that a larger beam size at the nozzle entrance means that a larger region of the quadrupoles is covered, leading to a greater difference in the fields experienced by particles in the beam center and the beam periphery which ultimately results in an enhanced focussing effect.

Table 3 compiles the results for the robustness simulations considering the effects of translational and rotational misalignment of the quadrupoles as well as field gradient errors. The simulations were carried out with the base beam model and an air gap of 10 cm using the quadrupole configurations listed in Table 2. The values in the columns Δ X, Δ Y, hFWHM and vFWHM are stated as *mean value* ± *standard deviation* calculated over 200 simulations. Additional percentage values given in parentheses refer to the magnitude of the standard deviation relative to the mean.

The results show that a misalignment of the quadrupoles mainly leads to a change in the spot position while it has a negligible effect on the spot size (standard deviations ≤0.5% except for one outlier of 1.7%). The absolute change in the spot position is very similar for the 100 MeV and 200 MeV beams and amounts to roughly ±2 mm for ΔX and ±3 mm for ΔY, independently of the considered spot position. Such errors, related to misalignment, would be systematic and constant from pulse to pulse. They could therefore be compensated by a calibration of the steering magnets during nozzle commissioning. The fact that greater variations were observed for ΔY than for ΔX may be due to the orientation of the focussing planes of the quadrupoles: Q1 which is further away from the target focussed vertically (y-direction) in all simulations while Q2 focussed horizontally.

Conversely, errors of the field gradient affect the spot size but leave the spot position unchanged (standard deviations <0.1%, not indicated in Table 3). Moreover, the absolute changes in FWHM are higher for the 200 MeV beams than for the 100 MeV beams. This is a consequence of the fact that the nominal quadrupole configuration at 100 MeV corresponds to the global minimum configuration (globally smallest Ω) whereas the 200 MeV configuration is slightly off the global minimum configuration which lies outside the considered range of $g_{1},g_{2}\leq 0.8$ T/m. The relative changes were between ≤2.8% at 100 MeV and ≤10.1% at 200 MeV.

### 3.2. Delivery of Conventional PBS

**Figure 3:**
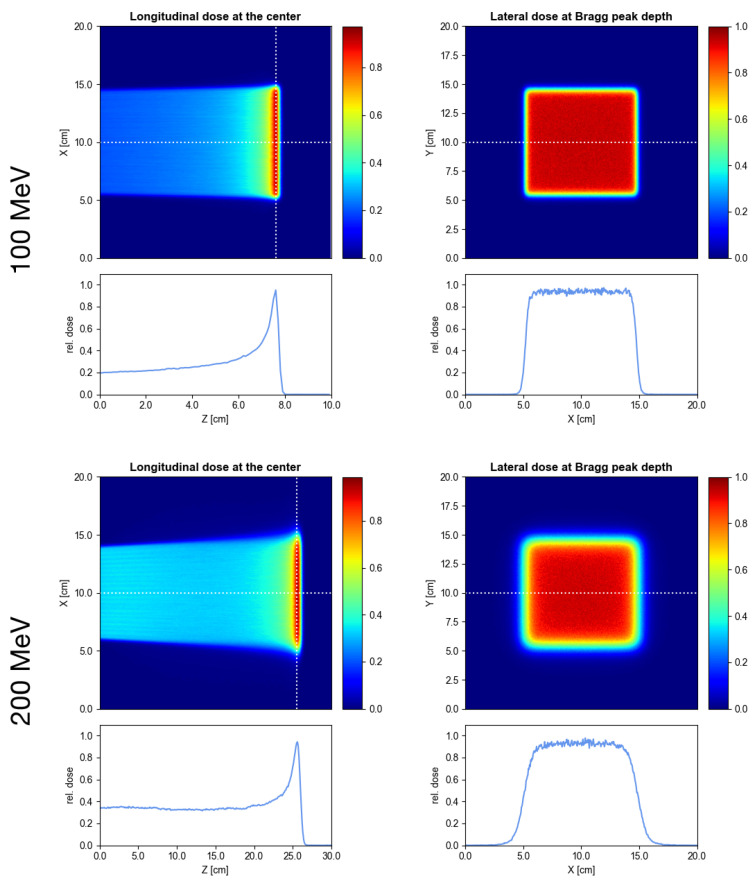
Lateral and longitudinal dose maps/profiles for an example pencil beam pattern at 100 MeV (**top**) and 200 MeV (**bottom**) assuming a 50 cm air gap. The vertical dotted line in the top left panel indicates the position in the Bragg peak where the lateral dose map was sampled.

Figure 3 shows the dose distributions in a water phantom of the 10×10$cm^{2}$ PBS field simulated for the 100 and 200 MeV beams. The air gap was 50 cm in both cases and the beam size at the phantom entrance (hFWHM/vFWHM) was 3.77±0.004/5.65±0.003 mm at 100 MeV and 4.31±0.001/4.04±0.002 mm at 200 MeV. Especially at the lower energy, these beam sizes are significantly smaller than those used in current PBS systems where the FWHM at 100 MeV usually lies between 10 and 30 mm [11,12,29,30,31,32].

A reduction of the beam size also allows to obtain a sharper lateral penumbra. From the lateral dose profiles at Bragg peak depth (bottom right panels in Figure 3), the 80–20% penumbrae (distance along the lateral fall-off from 80% to 20% of the mean dose in the plateau) were assessed to be 4.0±0.3 mm at 100 MeV and 10.0±0.3 mm at 200 MeV. These values are comparable to or even slightly smaller (by about 1–2 mm) than the penumbrae reported in the literature for similar irradiation patterns delivered with aperture-collimated PBS [33,34,35].

## 4. Discussion

Proton minibeam radiation therapy is a novel therapeutic approach which, in preclinical experiments, has already shown significant increases in the preservation of normal tissue [2,36,37] while providing equivalent or superior tumour control [3]. The optimal implementation of pMBRT should use magnetically focussed and scanned minibeams as this would allow it to maximise the irradiation efficiency and flexibility, decrease the contamination of secondary particles and yield a better spatial fractionation of the dose [8,9].

Previous experiments with magnetically focussed minibeams were already carried out at the ion-microprobe SNAKE in Munich, Germany [36,37,38]. However, beam energies there are currently limited to 20 MeV which is only suited for the irradiation of superficial lesions. An update of the facility is planned which would allow it to reach energies of up to 70 MeV, however the focus will remain on preclinical experiments and the irradiation of small animals [39]. Thus, the generation of magnetically focussed proton minibeams in a clinical context remains a challenge. The aim of this study was therefore to investigate the generation of such minibeams by evaluating our recently proposed new nozzle design [13] in combination with the LIGHT linear accelerator [15].

The results of the beam size minimisation study (Table 2) clearly show the suitability of this combination of the LIGHT accelerator and the new minibeam nozzle design for the generation of magnetically focussed proton minibeams at clinical beam energies. Moreover, they indicate that an air gap of 10 cm should be used in order to achieve minibeams also at lower energies. Air gaps ≤10 cm have already been used in previous proton therapy studies [34,35,40] and can therefore be considered realistic. In practice, the change between the 10 cm air gap needed for minibeams and a larger air gap for conventional PBS could be realised e.g., by moving the patient couch.

Due to the compact layout of the new nozzle design, the size of the air gap also plays an important role in the context of lateral scanning since a small gap also implies a short source-to-axis distance (SAD) which, for a given field size, determines the beam inclination angle. Conventional PBS systems typically have an SAD of around 2 m [41] and allow a maximum deviation from the centre by 15 cm, resulting in a maximum inclination angle of about 5 degrees. For the new nozzle design, an air gap of 10 cm corresponds to an SAD of 47.5–72.5 cm (measuring from the centre of SM2 and SM1, respectively). Assuming a slightly larger maximum beam inclination of 6-8 degrees would allow it to cover an area of 12×16$cm^{2}$ at the phantom entrance. Such a field size can be considered sufficient for many clinical cases. Moreover, even larger target volumes could be irradiated using techniques like field patching.

The evaluation of the different beam model variations demonstrates a good tolerance to variations in the incident beam parameters. Minibeam focussing can be achieved even for beams exhibiting emittances twice as high as those expected from simulations. The only exception to this might be a large increase in beam divergence which, however, could be mitigated through beam matching in the high energy beam transport section upstream of the nozzle. It should be noted that the emittance of the incoming beam alone does not provide sufficient information to predict whether magnetically focussed minibeams can be generated. Instead, the central parameter in this context is the divergence at the nozzle entrance which should be kept as low as possible but at least ≲0.5 mrad. These findings are in agreement with our previous results [13].

Beyond that, the robustness simulations show a sufficient tolerance to errors in quadrupole alignment and quadrupole field gradients. Considering typical quality assurance measurements performed at clinical centres [42,43], the practically tolerable limits are ±1–2 mm for the spot position and ±10–20% for the spot size, for energies ranging from 100 to 220 MeV. The spot size variations, which can be attributed to field errors, were observed to be well within this tolerance. The spot position error (amounting to ±2–3 mm), on the other hand, should be further reduced. However, as the errors only depend on misalignments, once the quadrupoles are installed, these errors are static and can therefore be corrected by applying an offset to the field strengths of the scanning magnets. All of these results indicate the robustness of the evaluated combination of the minibeam nozzle and linear accelerator.

It should be highlighted that an important aspect for the achievement of the observed minibeam sizes are the exceptionally small emittance and divergence of the beam provided by the LIGHT accelerator. Indeed, it would be very complicated if not impossible to achieve similar beam parameters in a cyclotron-based facility as the presence of a degrading energy selection system introduces too much emittance growth [14]. While synchrotrons may in principle deliver beams with similarly small emittances and divergences, in practice such beams usually exhibit nonetheless slightly larger emittances which, depending on the extraction method, may further be asymmetric.

Another advantage of the LIGHT design is the high pulse repetition frequency of 200 Hz which permits fast changing between energy layers. Moreover, the linac design allows to reach considerably higher dose rates (depending on the beam size and energy up to ∼50–1500 Gy/s at Bragg peak depth) which, together with the high irradiation efficiency of magnetically focussed minibeams [9], could open the door for a combination of pMBRT and FLASH therapy.

Finally, the results presented in Section 3.2 demonstrate that the combination of the linac and minibeam nozzle is also suitable for the delivery of conventional PBS fields. In order to switch between pMBRT and PBS mode, it would suffice to adjust the length of the air gap and the field in the quadrupole magnets. The pencil beam sizes considered in the simulations (between 3.8 and 5.7 mm FWHM) are considerably smaller than those typically used in modern PBS facilities. They may represent a good compromise between an enhanced sharpness of the dose distribution (due to the sharper penumbrae which otherwise can only be obtained with additional collimators [34,35,44]) and a reasonable irradiation time per energy layer. The LIGHT accelerator will feature a pulse repetition frequency of 200 Hz, meaning that a beam spot could be delivered every 5 ms. For the simulated 10×10 $cm^{2}$ fields, this would correspond to a time per layer of roughly 3.5 s for the 693 spots of the 100 MeV pattern and 2.2 s for the 441 spots of the 200 MeV pattern (cf. Section 2.3). The scanning order of the spot visits may be optimised so that the time needed for the displacement between spots could be accommodated within the 5 ms beam pulse periodicity. In summary, the possibility to deliver pencil beams with smaller beam sizes than conventional PBS facilities could improve the delivery of treatment plans and in particular the higher dose rate of magnetically focussed beams compared to collimated beams could further benefit applications in proton radiosurgery.

## 5. Conclusions

The aim of this study was to evaluate the performance of the new minibeam nozzle design in combination with the LIGHT accelerator. It has been demonstrated that, for an air gap of 10 cm, this combination is suitable for the generation of magnetically focussed minibeams at clinically relevant energies. Excellent beam sizes between 0.6 and 0.9 mm FWHM can be obtained, in particular due to the exceptionally small emittance and divergence of the beam provided by the linac.

The simulation of different beam models and quadrupole errors show a good tolerance for minibeam generation which further consolidates the aforementioned results and underlines the robustness of the proposed combination. The evaluation of example pencil beam patterns furthermore demonstrate the versatility of the combination of the LIGHT accelerator and minibeam nozzle which could also be used to perform standard PBS. Indeed, the possibility to decrease the size of the pencil beams beyond the limit of current PBS nozzles would allow it to obtain sharper penumbrae without the need for additional collimators which could further improve the delivery of treatment plans in conventional proton therapy as well as proton radiosurgery.

In conclusion, the results of this conceptual design study suggest that the combination of the LIGHT accelerator and the new minibeam nozzle could be a perfect match, allowing an optimal implementation of pMBRT and targeting at improved flexibility and efficiency in the dose delivery. Moreover, the high irradiation efficiency of magnetically focussed minibeams, compared to collimator-based techniques, would allow it to maximise the dose rate which could pave the way towards a combination of pMBRT and FLASH therapy. The next steps will be to perform a technical design study and ultimately the construction and testing of a nozzle prototype.

## Figures and Tables

**Table 1:** The different parametrisation of the virtual beam source. The emittances are given for reference and were not required for the simulation setup.

	*E*	ΔE	$\sigma_{x}$	$\sigma_{y}$	$\sigma_{x^{\prime }}$	$\sigma_{y^{\prime }}$	$r_{{\mathrm{xx}}^{\prime }}$	$r_{{\mathrm{yy}}^{\prime }}$	$\varepsilon_{x}$	$\varepsilon_{y}$
	[MeV]	[%]	[mm]	[mm]	[mrad]	[mrad]			[mm mrad]	[mm mrad]
**Base model**	100.5	0.22	0.30	0.45	0.54	0.53	−0.91	0.98	0.21	0.15
	199.7	0.15	0.24	0.47	0.20	0.45	0.19	0.97	0.15	0.16
**Var 1**	100.5	0.22	0.60	0.90	0.54	0.53	−0.91	0.98	0.42	0.30
(increased beam size)	199.7	0.15	0.48	0.94	0.20	0.45	0.19	0.97	0.30	0.32
**Var 2**	100.5	0.22	0.30	0.45	1.08	1.06	−0.91	0.98	0.42	0.30
(increased divergence)	199.7	0.15	0.24	0.47	0.40	0.90	0.19	0.97	0.30	0.32
**Var 3**	100.5	0.22	0.30	0.45	0.54	0.53	−0.56	0.92	0.42	0.29
(decreased correlation)	199.7	0.15	0.24	0.47	0.20	0.45	0.00	0.87	0.15	0.33
**Var 4**	100.5	0.22	0.38	0.57	0.68	0.67	−0.85	0.97	0.43	0.29
(combined variation)	199.7	0.15	0.30	0.59	0.25	0.57	0.00	0.95	0.24	0.33

**Table 2:** Results of the minimisation simulations stating the horizontal and vertical beam size (hFWHM and vFWHM) as well as the corresponding quadrupole field gradients ($g_{1}$ and $g_{2}$). The focussing plane configuration was the same in all cases with Q1 (Q2) focussing vertically (horizontally). Top: Results for the base beam model considering three different air gap lengths. Bottom: Results for the different beam model variations considering only the 10 cm air gap.

Beam size minimisation with unvaried base model					
*E* [MeV]	Air gap [cm]	hFWHM [mm]	vFWHM [mm]	$g_{1}$ [T/cm]	$g_{2}$ [T/cm]
100	10	$0.89_{-0.006}^{+0.002}$	$0.73_{-0.006}^{+0.002}$	0.608	0.496
	30	$1.81{}_{-0.007}^{+0.007}$	$1.67{}_{-0.009}^{+0.007}$	0.544	0.400
	50	$2.63{}_{-0.029}^{+0.019}$	$2.73{}_{-0.021}^{+0.019}$	0.432	0.256
200	10	$0.64_{-0.012}^{+0.002}$	$0.66_{-0.018}^{+0.002}$	0.800	0.752
	30	$1.04{}_{-0.008}^{+0.005}$	$0.99_{-0.013}^{+0.005}$	0.800	0.736
	50	$1.55{}_{-0.008}^{+0.005}$	$1.53{}_{-0.008}^{+0.005}$	0.800	0.736
**Beam size minimisation with model variations (air gap 10 cm)**					
*E* [MeV]	Beam model	hFWHM [mm]	vFWHM [mm]	$g_{1}$ [T/cm]	$g_{2}$ [T/cm]
100	var 1	$0.80_{-0.009}^{+0.002}$	$0.78_{-0.018}^{+0.002}$	0.544	0.496
	var 2	$1.55{}_{-0.008}^{+0.005}$	$0.92{}_{-0.018}^{+0.002}$	0.800	0.720
	var 3	$1.01{}_{-0.007}^{+0.005}$	$0.97_{-0.002}^{+0.002}$	0.672	0.640
	var 4	$1.08{}_{-0.007}^{+0.005}$	$0.82_{-0.006}^{+0.002}$	0.608	0.496
200	var 1	$0.54_{-0.012}^{+0.002}$	$0.52_{-0.012}^{+0.002}$	0.784	0.800
	var 2	$1.55{}_{-0.018}^{+0.005}$	$1.11{}_{-0.024}^{+0.005}$	0.800	0.576
	var 3	$0.59_{-0.006}^{+0.002}$	$0.85_{-0.012}^{+0.002}$	0.800	0.752
	var 4	$0.80_{-0.012}^{+0.002}$	$0.75_{-0.018}^{+0.002}$	0.800	0.720

**Table 3:** Average values and standard deviations for the simulation of quadrupole alignment errors (top) and field gradient errors (bottom).

*E* [MeV]	Spot	ΔX [mm]		ΔY [mm]		hFWHM [mm]		vFWHM [mm]	
**Translational and rotational alignment errors**									
100	center	0.08±2.16		0.15±3.31		0.88±0.003	(0.4%)	0.71±0.003	(0.4%)
	scan y	0.08±2.16		48.28±3.33	(6.9%)	0.88±0.003	(0.3%)	0.73±0.013	(1.7%)
	scan x	61.23±2.17	(3.5%)	0.15±3.32		0.90±0.004	(0.4%)	0.71±0.002	(0.4%)
	scan xy	61.42±2.18	(3.5%)	48.45±3.34	(6.9%)	0.91±0.003	(0.4%)	0.73±0.004	(0.5%)
200	center	−0.21±1.95		0.09±3.03		0.62±0.002	(0.3%)	0.66±0.002	(0.3%)
	scan y	−0.22±1.95		67.00±3.07	(4.6%)	0.62±0.002	(0.4%)	0.67±0.003	(0.4%)
	scan x	84.70±1.97	(2.3%)	0.09±3.04		0.64±0.002	(0.4%)	0.66±0.003	(0.4%)
	scan xy	85.21±1.98	(2.3%)	67.47±3.08	(4.6%)	0.65±0.003	(0.4%)	0.67±0.003	(0.5%)
**Field gradient errors**									
100	center	0.00±0.001		0.00±0.001		0.88±0.008	(1.0%)	0.72±0.020	(2.8%)
	scan y	0.00±0.001		48.13±0.001		0.89±0.009	(1.0%)	0.74±0.021	(2.8%)
	scan x	61.15±0.001		0.00±0.001		0.91±0.009	(1.0%)	0.72±0.020	(2.8%)
	scan xy	61.34±0.002		48.31±0.001		0.91±0.009	(1.0%)	0.75±0.021	(2.8%)
200	center	0.00±0.001		0.00±0.001		0.62±0.029	(4.7%)	0.67±0.068	(10.2%)
	scan y	0.00±0.001		66.92±0.001		0.62±0.029	(4.7%)	0.68±0.066	(9.7%)
	scan x	84.91±0.001		0.00±0.001		0.64±0.028	(4.4%)	0.67±0.068	(10.1%)
	scan xy	85.42±0.001		67.38±0.001		0.65±0.028	(4.4%)	0.68±0.066	(9.6%)

## Data Availability

The data presented in this study are available upon reasonable request to the corresponding author.

## Abbreviations

The following abbreviations are used in this manuscript:

CCL	cell coupled linac
FWHM	full width at half maximum
hFWHM	horizontal full width at half maximum
LIGHT	Linac For Image Guided Hadron Therapy
linac	linear accelerator
PBS	pencil beam scanning
pMBRT	proton minibeam radiation therapy
RFQ	radio frequency quadrupole
SAD	source-to-axis distance
SCDTL	side coupled drift tube linac
vFWHM	vertical full width at half maximum
